# Distinct Roles Between Eotaxin 1 and Eotaxin 2 in Asthmatic Airways

**DOI:** 10.1002/clt2.70077

**Published:** 2025-07-12

**Authors:** Soyoon Sim, Eun‐mi Yang, Yoo Seob Shin, Seon Beom Kim, Youngwoo Choi, Hae‐Sim Park

**Affiliations:** ^1^ Department of Allergy and Clinical Immunology Ajou University School of Medicine Suwon Korea; ^2^ Department of Food Science and Technology Pusan National University Miryang Korea; ^3^ Institute for Future Earth Pusan National University Busan Korea; ^4^ Department of Biomaterials Science (BK21 FOUR Program) College of Natural Resources and Life Science Life and Industry Convergence Research Institute Pusan National University Miryang Korea

**Keywords:** airway epithelial cells, airway remodeling, asthma, eosinophils, eotaxin, neutrophils

## Abstract

**Background:**

Eotaxins (EOTs), primarily expressed in airway epithelial cells (AECs), act as chemoattractants for eosinophils in asthma pathogenesis. Recent studies have suggested that EOTs have additional functions beyond chemotaxis. However, the distinct roles of EOTs remain incompletely understood.

**Methods:**

Serum EOT1, EOT2, myeloperoxidase (MPO), matrix metalloproteinase‐9 (MMP‐9), and tissue inhibitor of metalloproteinase‐1 (TIMP‐1) levels were evaluated by ELISA and serum eosinophil cationic protein (ECP) levels were measured by ImmunoCAP in 79 adult asthmatics. Clinical characteristics were analyzed by inflammatory phenotype, disease severity, and serum EOT1/EOT2 levels. The functions of EOTs were investigated in vitro and ex vivo. For in vivo, EOT1 and EOT2 were intranasally administered to ovalbumin/lipopolysaccharide‐induced asthmatic mice (BALB/c). To assess neutralization effects, anti‐EOT1 or anti‐EOT2 antibodies were intranasally administered.

**Results:**

Serum EOT1 and EOT2 levels were higher in patients with severe asthma (SA) than in those with non‐SA. Serum EOT1 levels were associated with increased blood/sputum eosinophil counts and serum ECP levels, but not significantly correlated with FEV_1_ (%) values. In contrast, serum EOT2 levels are correlated with higher serum MPO, MMP‐9, and TIMP‐1 levels but lower FEV_1_ (%). In asthmatic mice, EOT1 increased eosinophil counts and IL‐5 production, whereas EOT2 induced CXCL1 and MMP‐9 production, junctional dysfunction and epithelial‐to‐mesenchymal transition in the lungs, which were attenuated by neutralizing EOTs using each antibody.

**Conclusion:**

EOT1 promotes T2/eosinophilic inflammation, whereas EOT2 accelerates airway remodeling and lung function decline by activating neutrophils, providing a new insight into the distinct roles of EOTs in the pathogenesis of SA.

## 1 Introduction

Asthma is a chronic airway inflammatory disease with heterogenous phenotypes driven by complex immunologic mechanisms. The predominant features in asthmatic patients are sputum and blood eosinophilia, which are closely mediated by type 2 (T2) immune responses involving increased production of T2 cytokines (IL‐4, IL‐5, and IL‐13) in T cells, as well as IgE in B cells, basophils, and mast cells. Thus, eosinophils serve as key effector cells in asthma pathogenesis and function as indicators for determining T2/eosinophilic phenotype and disease severity [1].

As more than 50% of patients with severe asthma (SA) exhibit T2/eosinophilic inflammation, the development of biologics targeting T2 immune responses has allowed most asthmatic patients to achieve disease control under recommended guidelines. However, patients with SA still suffer from persistent symptoms and frequent exacerbation despite receiving standardized treatment, including inhaled corticosteroid with additional controllers or oral corticosteroid [2]. In particular, neutrophilic inflammation is often found in the airways of patients with SA or steroid‐refractory asthma, indicating that neutrophils can contribute to disease severity and steroid resistance [3, 4]. Furthermore, neutrophils promote airway remodeling by releasing various granule proteins and mediators, including neutrophil elastase (NE), myeloperoxidase (MPO), and matrix metalloproteinase‐9 (MMP‐9), which induce subepithelial fibrosis, tight junction disruption, epithelial‐to‐mesenchymal transition (EMT), and extracellular matrix (ECM) deposition, ultimately leading to rapid airflow obstruction and lung function decline [5, 6, 7].

Eotaxins (EOTs) function as chemoattractants for eosinophils, facilitating their recruitment and activation in asthmatic airways by binding to CC chemokine receptor 3 (CCR3). EOTs are mainly released by airway epithelial cells (AECs), airway smooth muscle cells, and fibroblasts in response to IL‐4 and IL‐13 stimulation. EOTs are classified into 3 subfamilies according to their different chromosomal gene loci [8]. Although the distinct roles of each EOT subtype remain unclear, recent findings have suggested that EOT1 mediates the early recruitment of eosinophils to asthmatic airways, whereas EOT2 and EOT3 contribute to the sustained presence of airway eosinophilia after allergen challenges [9]. To date, the chemotactic roles of EOTs have been considered selective for eosinophils and basophils. However, several studies have indicated that infiltrated neutrophils acquire CCR3 expression on their surface during inflammation [10, 11, 12]. Moreover, intradermal injection of EOT2 induced neutrophil recruitment and granule secretion in the skin of atopic volunteers [10]. In addition, an in vivo study demonstrated that CCR3 antagonist reduced neutrophil infiltration and activation, represented by increased CD11b expression and tumor necrosis factor‐α (TNF‐*α*) and chemokine C‐X‐C ligand 1 (CXCL1) production, in the injured or infected murine lungs by lipopolysaccharide (LPS) or influenza A [13]. Furthermore, EOT2 has been reported to promote collagen production and ECM deposition in human lung fibroblasts, suggesting that EOT2 may contribute to airway remodeling and lung fibrosis in asthmatic patients [14]. Based on these findings, we hypothesized that the blockade of EOT2 from binding CCR3 can be a potential therapeutic target for suppressing neutrophilic inflammation and fibrotic remodeling in asthmatic airways. Therefore, this study aimed to investigate the association between EOTs and neutrophils or eosinophils in the pathogenesis of SA by examining (1) the clinical significance of EOTs in relation to asthma phenotypes and severity and (2) the functional roles of EOT1 and EOT2 on airway inflammation through in vitro, in vivo, and ex vivo analyses.

## 2 Methods

### 2.1 Patient Recruitment and Clinical Parameters

This study was approved by the Institutional Review Board of Ajou University Hospital (AJIRB‐GEN‐SMP‐13‐108 and AJIRB‐BMR‐SUR‐15‐498). Written informed consents were obtained from all the study subjects at the time of recruitment. Serum samples were collected at the initial diagnosis and stored at −70°C for further analysis. In this study, 79 adult asthmatic patients aged ≥ 20 years were enrolled. All patients were diagnosed by allergy specialists based on clinical histories in accordance with the Global Initiative for Asthma (GINA) guidelines [15]. Asthmatic patients were classified into eosinophilic asthma (EA) and neutrophilic asthma (NA) groups based on sputum eosinophils (≥ 3%) and neutrophils (≥ 65%) percentage, as previously described. SA was defined according to the guidelines of the European Respiratory Society and American Thoracic Society [16]. Atopy was defined as the presence of at least one positive result in skin prick tests for common inhaled allergens (Bencard, Bradford, UK). The serum total IgE and eosinophil cationic protein (ECP) were measured using the ImmunoCAP system (ThermoFisher Scientific, Waltham, CA, USA). The serum levels of MMP‐9, tissue inhibitor of metalloproteinase‐1 (TIMP‐1), EOT1, EOT2, and MPO were measured using respective ELISA kits (R&D Systems, Minneapolis, MN, USA) according to the manufacturer's recommendations. To determine the cut‐off value for the high‐ and low‐EOT1 or ‐EOT2 groups, 95% confidence intervals of mean were used. The intervals obtained by adding standard deviation (SD) from means showed the chance of a test value falling outside less than 5%. The higher limit of this interval was considered the cut‐off values.

### 2.2 Human Peripheral Blood Cell Isolation

Peripheral blood from asthmatic patients was collected in a BD Vacutainer tube containing acid citrate dextrose solution (BD biosciences, Franklin Lakes, NJ, USA) and layered onto Lymphoprep solution (Axis‐Sield, Oslo, Norway). After centrifugation at 879 × g for 25 min at 20°C, the layer containing granulocytes was separated in Hank's balanced salt solution buffer with 2 mmol/L ethylenediaminetetraacetic acid and 2% dextran for 20 min. Upon removal of red blood cells, eosinophils and neutrophils were isolated via magnetic activated cell sorting (Miltenyi Biotec, Bergisch Gladbach, Germany) according to the manufacturer's recommendations. Isolated neutrophils from patients with non‐SA (NSA) and SA were treated with EOT1 or EOT2 (100 ng/mL) under stimulation with LPS (100 ng/mL). The concentrations of MPO and MMP‐9 in the supernatants were measured using respective ELISA kit (R&D Systems) according to the manufacturer's recommendations.

### 2.3 Stimulation of AEC

BEAS‐2B cells (American Type Culture Collection, Manassas, VA, USA) were cultured in RPMI‐1640 medium supplemented with 10% fetal bovine serum, 100 IU/mL penicillin, and 50 μg/mL streptomycin (ThermoFisher Scientific) and grown at 37°C in humidified air with 5% CO_2_. Then, the cells were seeded in 6‐well plates (2 × 10^5^) and co‐cultured with eosinophils or neutrophils (1 × 10^5^) for 24 h. The levels of EOT1 and EOT2 in culture supernatants were quantified using the respective ELISA kit according to the manufacturer's recommendations. To investigate the effect of blocking EOTs on AECs, BEAS‐2B cells (2 × 10^5^) seeded in 6‐well plates were treated with anti‐EOT1 or anti‐EOT2 antibodies (100 ng/mL) for 24 h in the presence of peripheral neutrophils (1 × 10^5^) from patients with SA. The concentrations of MMP‐9 and TIMP‐1 in culture supernatants were measured using the respective ELISA kit (R&D Systems) according to the manufacturer's recommendations.

### 2.4 Mouse Experiment and Evaluation

Animal studies were approved by the Institutional Animal Care and Use Committee of Ajou University (IACUC‐2024‐0016). All experiments were conducted according to the Guidelines for the Care and Use of Laboratory Animals suggested by Animal and Plant Quarantine Agency, Ministry of Agriculture, Food and Rural Affairs, Republic of Korea. To establish asthmatic mouse model, female 6‐weeks‐old BALB/c mice (Orient BIO, Seongnam, Korea) were intraperitonially injected with 75 μg of ovalbumin (OVA), 10 μg of LPS (Sigma‐Aldrich, St. Louis, MO, USA), and 2 mg aluminum hydroxide (Alum; ThermoFisher Scientific) for sensitization on days 0 and 14. Sensitized mice then received intranasal injection with 50 μg OVA and 10 ng LPS in 20 μL of PBS on days 21, 23, 25, 27, 29, and 31 as challenge exposures. To investigate the distinct roles of EOTs in NA, mice were intranasally treated with EOT1 or EOT2 (1 μg/mL) during the challenges phase. To compare the effects of EOT antagonists with corticosteroid treatment, anti‐EOT1 antibody, anti‐EOT2 antibody (1 μg/mL), or dexamethasone (Dex; 1 mg/kg) were intranasally administered simultaneously with the challenge exposures. Airway hyperresponsiveness (AHR) to inhaled methacholine (Sigma‐Aldrich) was assessed using the flexiVent System (SCIREQ, Montreal, Canada). The counts of immune cells in bronchoalveolar lavage fluid (BALF) were evaluated using diff‐Quick staining (Dada Behring, Dudingen, Switzerland). The concentrations of CXCL1, IL‐5, IL‐13, and MMP‐9 in BALF were measured by using the respective ELISA kits (R&D Systems) according to the manufacturer's recommendations. For histological analysis, lung tissues were stained with hematoxylin and eosin (H&E), periodic acid‐Schiff (PAS), and Masson's trichrome (MT).

### 2.5 Western Blot Analysis

The antibodies used were as follows: MMP‐9, TIMP‐1, *α*‐smooth muscle actin (α‐SMA), E‐cadherin, vimentin (Cell Signaling Technology, Danvers, MA, USA), occludin, claudin‐1, and actin (Santa Cruz, Dallas, TX, USA) for both human and mouse samples.

### 2.6 Statistical Analysis

All statistical analyses were performed using IBM SPSS software, version 26.0 (IBM Corp., Armonk, NY, USA). Graphs were generated using GraphPad Prism 8.0 software (GraphPad Inc., San Diego, CA, USA).

## 3 Results

### 3.1 Comparison of Serum EOT1 and EOT2 Levels According to Asthma Phenotypes

When patients were classified into EA and NA groups, no significant differences were found in sex, atopy rates or lung functions. However, patients with EA exhibited higher total IgE levels and blood/sputum eosinophil counts, whereas those with NA had older age and higher sputum neutrophil counts (*p* < 0.05 for all). Notably, serum EOT1 levels were significantly higher in patients with EA than in those with NA (*p* = 0.010); serum MMP‐9, TIMP‐1, and EOT2 levels did not statistically differ between the 2 groups. When asthmatic patients were classified according to disease severity, no significant differences were observed in age, sex, atopy rates, PC20 methacholine values, or sputum eosinophil and neutrophil counts. However, patients with SA showed lower forced expiratory volume in 1 s (FEV_1_) (%) values and serum total IgE levels but higher blood eosinophil counts and serum MMP‐9 and TIMP‐1 levels compared to those with NSA (*p* < 0.05 for all). Furthermore, serum EOT1 and EOT2 levels were significantly higher in patients with SA than in those with NSA (*p* < 0.05; Table 1), suggesting that elevated serum levels of EOT1 and EOT2 are associated with asthma severity.

**TABLE 1:** Demographic data of asthmatic patients according to asthma phenotypes.

Variables	Eosinophilic (*n* = 44)	Neutrophilic (*n* = 35)	*p* value	SA (*n* = 28)	NSA (*n* = 51)	*p* value
Age (year)	42.0 ± 13.7	51.4 ± 15.8	0.006	47.4 ± 15.1	45.5 ± 15.6	0.600
Female sex (%)	54.5	71.4	0.387	67.9	58.8	0.429
Atopy (%)	48.8	55.2	0.213	38.5	59.1	0.152
FEV1 (%)	83.6 ± 21.3	85.7 ± 18.3	0.701	73.5 ± 20.9	90.4 ± 16.8	0.002
PC20 methacholine (mg/mL)	9.7 ± 10.8	10.3 ± 10.7	0.831	8.7 ± 10.7	10.5 ± 10.8	0.586
Total IgE (kU/L)	555.7 ± 901.6	225.5 ± 310.4	0.033	227.5 ± 244.8	512.4 ± 867.4	0.040
Blood eosinophils (/μL)	559.3 ± 427.0	205.2 ± 280.4	0.001	566.6 ± 462.8	339.1 ± 368.9	0.045
Sputum eosinophil (%)	55.9 ± 35.5	0.2 ± 0.6	0.001	42.5 ± 44.0	30.2 ± 36.1	0.329
Sputum neutrophil (%)	39.8 ± 33.0	73.9 ± 28.3	0.001	46.5 ± 33.0	57.6 ± 36.1	0.331
MMP‐9 (ng/mL)	242.4 ± 189.7	271.2 ± 323.5	0.622	372.8 ± 346.6	190.5 ± 160.0	0.013
TIMP‐1 (ng/mL)	187.4 ± 111.0	172.6 ± 73.2	0.481	207.9 ± 96.6	164.6 ± 86.8	0.049
EOT1 (pg/mL)	65.5 ± 61.4	6.8 ± 23.2	0.001	61.3 ± 67.9	27.5 ± 45.3	0.010
EOT2 (pg/mL)	878.0 ± 786.2	781.6 ± 799.3	0.592	1280.9 ± 1000.5	590.7 ± 506.9	0.002
SA (%)	45.5	22.9	0.037	100.0	0.0	NA

*Note:* Values are given as *n* (%) for categorical variables and as mean ± SD for continuous variables. *p* values were obtained by Pearson's chi‐squared test for categorical variables and Student's t test for continuous variables.

Abbreviations: Eos, eosinophil; EOT1, eotaxin 1; EOT2, eotaxin 2; FEV_1_, forced expiratory volume in 1 s; IgE, immunoglobulin E; methacholine PC20, the provocative concentration of methacholine required to cause a 20% fall in FEV_1_; MMP‐9, matrix metallopeptidase 9; NA, not available; Neu, neutrophil; NSA, non‐severe asthma; SA, severe asthma; TEC, total eosinophil counts; TIMP‐1, tissue inhibitor of metalloproteinase.

### 3.2 Association Between EOT1 and Eosinophilic Inflammation in Asthmatic Patients

**FIGURE 1:**
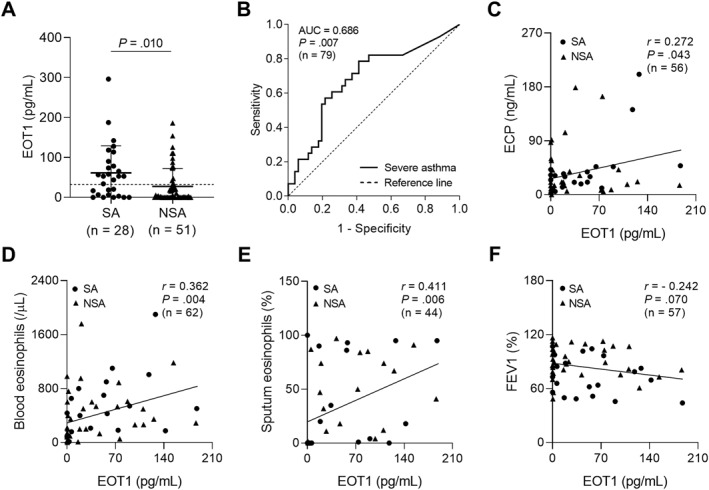
Association between serum EOT1 levels and eosinophilic inflammation in the study subjects. (A) The comparison of serum EOT1 levels between patients with SA and those with NSA. Data are represented as the means ± SDs. *p* values were obtained by students' *t* test. (B) A receiver operator curve analysis for EOT1 in asthmatic patients. Correlations between serum EOT1 levels and (C) serum ECP, (D) blood eosinophil counts, (E) sputum eosinophil percentages, and (F) FEV_1_ (%) values. Data are presented as Pearson correlation coefficient *r* (*p* value). ECP, eosinophil cationic protein; EOT1, eotaxin 1; FEV_1_, forced expiratory volume in 1 s; NSA, non‐severe asthma; SA, severe asthma.

Given that serum EOT1 levels were significantly higher in patients with EA than in those with NA (*p* = 0.001; Table 1), the contribution of EOT1 to eosinophilic inflammation was investigated. As a result, serum EOT1 levels were also higher in patients with SA than in those with NSA (*p* = 0.010; Figure 1A), suggesting its potential as a biomarker for distinguishing patients with SA from those with NSA (AUC = 0.686, *p* = 0.007; Figure 1B). Additionally, serum EOT1 levels were positively correlated with serum ECP levels and blood and sputum eosinophil counts (*r* = 0.272, *p* = 0.043, *r* = 0.362, *p* = 0.004, and *r* = 0.411, *p* = 0.006, respectively; Figure 1C‒E) but negatively correlated with FEV_1_ (%) values (*r* = ‐ 0.242, *p* = 0.070; Figure 1F). These findings suggest that EOT1 contribute to asthma severity by promoting eosinophilic airway inflammation albeit no significant association with lung function decline was observed.

### 3.3 Association Between EOT2 and Airway Remodeling in Asthmatic Patients

**FIGURE 2:**
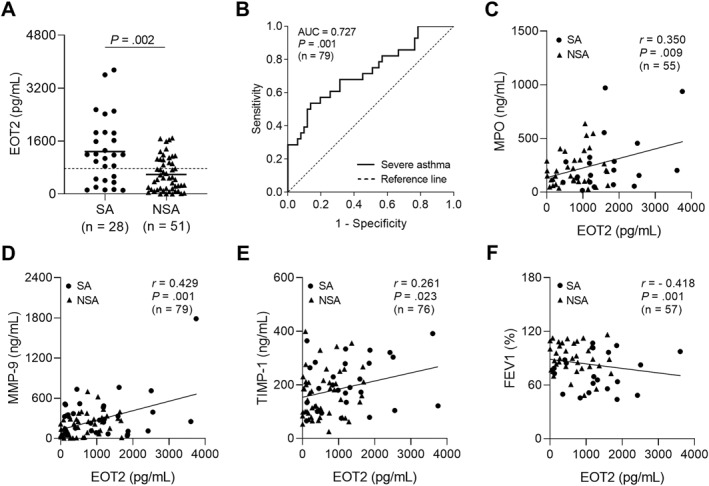
Association between serum EOT2 levels and airway remodeling in the study subject. (A) The comparison of serum EOT2 between patients with SA and those with NSA. Data are represented as the means ± SDs. *p* values were obtained by students' *t* test. (B) A receiver operator curve analysis for EOT2 in asthmatic patients. Correlations between serum EOT2 levels and (C) serum MPO, (D) MMP‐9, and (E) TIMP‐1 levels, and (F) FEV1 (%) values. Data are presented as Pearson correlation coefficient *r* (*p* value). EOT2, eotaxin 2; MMP‐9, matrix metallopeptidase 9; MPO, myeloperoxidase; TIMP‐1, tissue inhibitor of metalloproteinase.

When asthmatic patients were classified according to disease severity, serum EOT2 levels were significantly higher in patients with SA than in those with NA (*p* = 0.002; Figure 2A). Notably, receiver operator characteristics curve analysis demonstrated that serum EOT2 levels could serve as an effective discriminator between patients with SA and those with NSA (AUC = 0.727, *p* = 001; Figure 2B). Furthermore, serum EOT2 levels showed positive correlations with serum MPO, MMP‐9, and TIMP‐1 levels (*r* = 0.350, *p* = 0.009, *r* = 0.0.429, *p* = 0.001, and *r* = 0.261, *p* = 0.023, respectively; Figure 2C‒E) but a negative correlation with FEV_1_ (%) values (*r* = ‐ 0.418, *p* = 0.001; Figure 2F). These data suggest that EOT2 contributes to neutrophilic activation and airway remodeling in the pathogenesis of SA.

### 3.4 Differential Roles of EOT1 and EOT2 in the Development of SA

When asthmatic patients were classified into the high‐ and low‐EOT groups based on respective cut‐off values (19.29 pg/mL for EOT1 and 743.55 pg/mL for EOT2), no significant differences were observed in sex, atopy rates, PC20 methacholine values, serum total IgE levels or sputum neutrophil counts between the 2 groups. However, patients in the low‐EOT1 group were older than those in the high‐EOT1 group (*p* = 0.012). Additionally, blood or sputum eosinophil counts, as well as serum ECP levels, were significantly higher in the high‐EOT1 group than in the low‐EOT1 group (*p* = 0.001, *p* = 0.003, and *p* = 0.041, respectively). The prevalence of SA was also higher in the high‐EOT1 group than in the low‐EOT1 group (*p* = 0.008). Although sputum neutrophils count did not differ between the high‐ and low‐EOT2 groups, significantly lower FEV_1_ (%) values were noted in the high‐EOT2 group than in the low‐EOT2 group (*p* = 0.001). Furthermore, the levels of serum MPO, MMP‐9, and TIMP‐1 were significantly higher in the high‐EOT2 group than in the low‐EOT2 group (*p* = 0.042, *p* = 0.048, and *p* = 0.022, respectively). The proportion of patients with SA was also greater in the high‐EOT2 group than in the low‐EOT2 group (*p* = 0.006; Table 2). These findings suggest that EOT1 is associated with eosinophil infiltration and activation, whereas EOT2 contributes to neutrophil activation and lung function decline, ultimately promoting progression to SA.

**TABLE 2:** Comparison of clinical and laboratory findings in asthmatic patients according to the cut‐off values of EOT1 (19.29 pg/mL) or EOT2 (743.55 pg/mL).

Variables	EOT1‐high (*n* = 35)	EOT1‐low (*n* = 44)	*p* value	EOT2‐high (*n* = 37)	EOT2‐low (*n* = 42)	*p* value
Age (year)	41.3 ± 13.9	50.0 ± 15.5	0.012	47.7 ± 12.7	44.8 ± 17.4	0.402
Female sex (%)	60.0	63.6	0.741	64.9	59.5	0.625
Atopy (%)	45.5	56.8	0.359	48.6	54.5	0.592
FEV1 (%)	81.9 ± 21.3	86.8 ± 18.6	0.361	75.9 ± 19.2	96.3 ± 14.3	0.001
PC20 methacholine (mg/mL)	10.9 ± 11.5	9.2 ± 10.1	0.558	8.4 ± 10.4	11.4 ± 10.9	0.307
Total IgE (kU/L)	546.6 ± 818.4	299.1 ± 618.5	0.146	304.9 ± 636.3	520.0 ± 793.6	0.205
Blood eosinophils (/μL)	616.0 ± 460.7	231.5 ± 255.5	0.001	367.7 ± 286.4	442.6 ± 503.5	0.475
Sputum eosinophil (%)	50.7 ± 36.7	16.5 ± 33.6	0.003	28.4 ± 37.4	43.9 ± 40.4	0.204
Sputum neutrophil (%)	46.3 ± 33.6	62.4 ± 35.7	0.126	57.2 ± 33.0	50.4 ± 38.1	0.520
ECP (ng/mL)	50.4 ± 57.0	24.8 ± 23.5	0.041	33.7 ± 36.7	40.1 ± 51.7	0.591
MPO (ng/mL)	231.6 ± 225.3	234.3 ± 192.0	0.961	268.6 ± 239.3	170.9 ± 105.3	0.042
MMP‐9 (ng/mL)	282.6 ± 193.8	233.3 ± 297.2	0.400	318.1 ± 314.8	199.7 ± 176.7	0.048
TIMP‐1 (ng/mL)	180.0 ± 193.8	180.0 ± 78.8	0.995	205.3 ± 89.1	157.1 ± 90.0	0.022
SA (%)	51.4	22.7	0.008	51.4	21.4	0.006

*Note:* Values are given as *n* (%) for categorical variables and as mean ± SD for continuous variables. *p* values were obtained by Pearson's chi‐squared test for categorical variables and Student's t test for continuous variables.

Abbreviations: ECP, eosinophil cationic protein; eos, eosinophil; EOT1, eotaxin 1; EOT2, eotaxin 2; FEV_1_, forced expiratory volume in 1 s; IgE, immunoglobulin E; methacholine PC20, the provocative concentration of methacholine required to cause a 20% fall in FEV1; MMP‐9, matrix metallopeptidase 9; MPO, myeloperoxidase; neu, neutrophil; NSA, non‐severe asthma; SA, severe asthma; TEC, total eosinophil counts; TIMP‐1, tissue inhibitor of metalloproteinase.

### 3.5 Distinct Functions of EOT1 and EOT2 in AECs and Neutrophils

**FIGURE 3:**
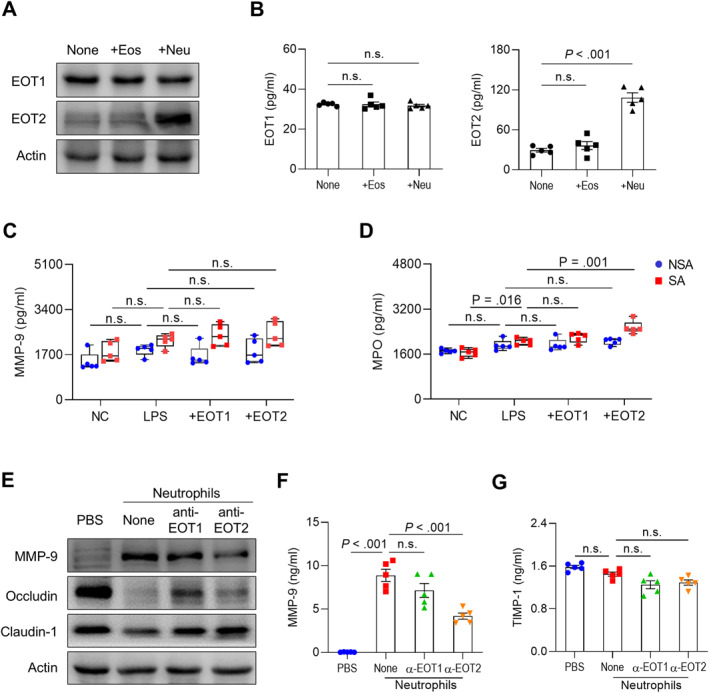
Distinct function of EOT1 and EOT2 in AECs and neutrophils. (A) Expressions and (B) concentrations of EOT1 and EOT2 in BEAS‐2B cells. Data are presented as box plots, *n* = 5. *p* values were obtained by one‐way ANOVA with Bonferroni's post hoc test. The levels of (C) MMP‐9 and (D) MPO in neutrophils isolated from patients with SA or NSA. Data are presented as box plots, *n* = 5. *p* values were obtained by Student's *t*‐test. (E) Expressions of MMP‐9 and tight junction proteins in BEAS‐2B cells. Concentrations of (F) MMP‐9 and (G) TIMP‐1 in the supernatants of BEAS‐2B cells. Data are presented as box plots, *n* = 5. *p* values were obtained by one‐way ANOVA with Bonferroni's post hoc test. Eos, eosinophil; LPS, lipopolysaccharide; Neu, neutrophil.

To investigate the distinct functions of EOTs in the pathogenesis of SA, AECs were co‐cultured with eosinophils or neutrophils. As a result, the production and expression of EOT2 were significantly increased in AECs co‐cultured with neutrophils, whereas EOT1 levels remained unchanged (Figure 3A and B). Based on these findings, isolated neutrophils from patients with SA and NSA were treated with EOT1 or EOT2 under LPS stimulation. As a result, neither EOT1 nor EOT2 directly induced MMP‐9 production in neutrophils (Figure 3C). However, in neutrophils from patients with SA, EOT2—but not EOT1—further enhanced LPS‐induced MPO secretion whereas no differences were observed in neutrophils from patients with NSA (Figure 3D). These results suggest that EOT2 activates neutrophils by increasing MPO secretion without affecting MMP‐9 production. To block EOT binding to CCR3 on neutrophils, AECs were treated with anti‐EOT1 or anti‐EOT2 antibodies in the presence of neutrophils isolated from patients with SA. As a result, neutrophils increased MMP‐9 expression and degraded tight junction proteins in AECs, which were partially reversed by anti‐EOT1 and ‐EOT2 antibodies (Figure 3E). Notably, anti‐EOT2 antibody reduced MMP‐9 levels but did not affect TIMP‐1 levels in the supernatants of AECs co‐cultured with neutrophils (Figure 3F and G). These data suggest that elevated EOT2 production in patients with SA contributes to neutrophil activation, leading to increased MMP‐9 production and tight junction disruption in asthmatic airways.

### 3.6 Contribution of EOTs in Airway Inflammation and Remodeling in Asthmatic Mice

**FIGURE 4:**
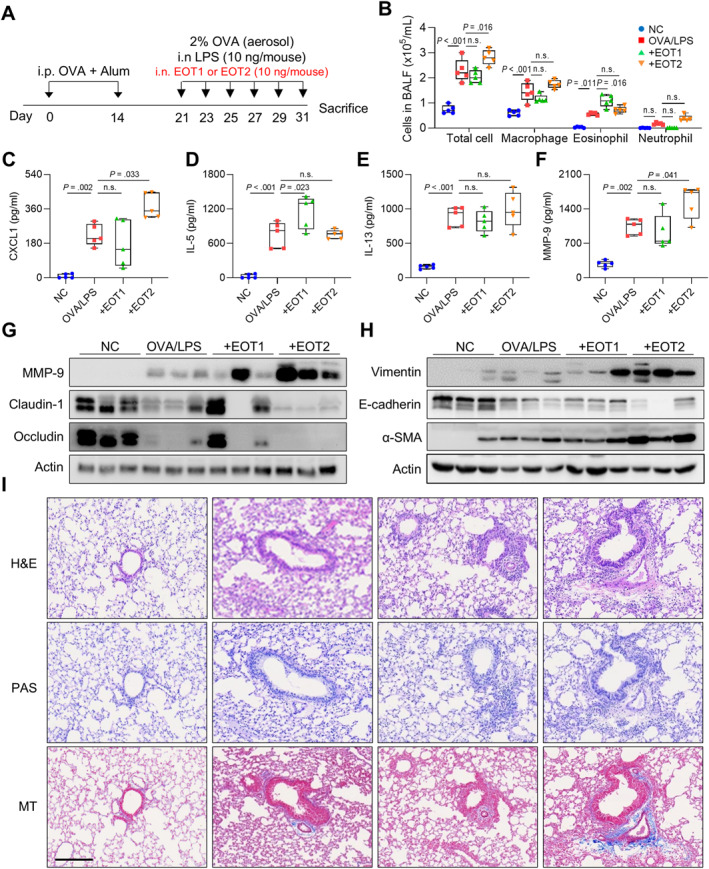
Distinct function of eotaxins in airway inflammation and remodeling in asthmatic mice. (A) Experimental schedules. (B) Differential cell counts. The levels of (C) CXCL1, (D) IL‐5, (E) IL‐13, and (F) MMP‐9 in BALF. Data are presented as box plots, *n* = 5. *p* values were obtained by one‐way ANOVA with Bonferroni's post hoc test. Expressions of (G) MMP‐9 and tight junctions and (H) adherent junctions in the lung tissues. (I) Histological analysis of lung tissues stained with H&E, PAS, and MT. Scale bar, 200 μm. Alum, aluminum hydroxide; *α*‐SMA, alpha‐smooth muscle actin; CXCL1, chemokine (C‐X‐C motif) ligand 1; Dex, dexamethasone’ H&E, hematoxylin and eosin; LPS, lipopolysaccharide; MT, Masson's trichrome; NC, normal control; OVA, ovalbumin; PAS, periodic acid‐Schiff.

To investigate differential functions of EOTs in asthma pathogenesis, asthmatic mice were intranasally injected with EOT1 or EOT2, as shown in Figure 4A. As a result, asthmatic mice showed increased total cell, macrophage, and eosinophil counts in BALF. Additionally, EOT1 significantly increased eosinophilia in the BALF, whereas EOT2 increased total cell counts. However, no differences were observed in macrophage and neutrophil counts (Figure 4B). Furthermore, EOT1 significantly increased IL‐5 levels whereas EOT2 elevated CXCL1 and MMP‐9 levels in BALF (Figure 4C–F). Notably, EOT2 enhanced MMP‐9 expression and depleted tight junction proteins, such as claudin‐1 and occludin in lung tissues. Additionally, EOT2 promoted EMT and fibrosis by increasing vimentin and *α*‐SMA expressions while decreasing E‐cadherin expression. In contrast, EOT1 did not alter any markers associated with airway remodeling (Figure 4G and H). Immunohistochemical analysis showed no notable changes in immune cell infiltrations after EOT1 or EOT2 treatment. However, epithelium thickness and collagen deposition were increased by EOT2 but not by EOT1 (Figure 4I). These findings suggest that EOT1 promotes airway inflammation by increasing eosinophil counts and IL‐5 production whereas EOT2 accelerates fibrotic remodeling by inducing EMT and tight junction disruption in asthmatic airways.

### 3.7 Regulation of Airway Inflammation and Remodeling by Neutralizing EOTs in Asthmatic Mice

**FIGURE 5:**
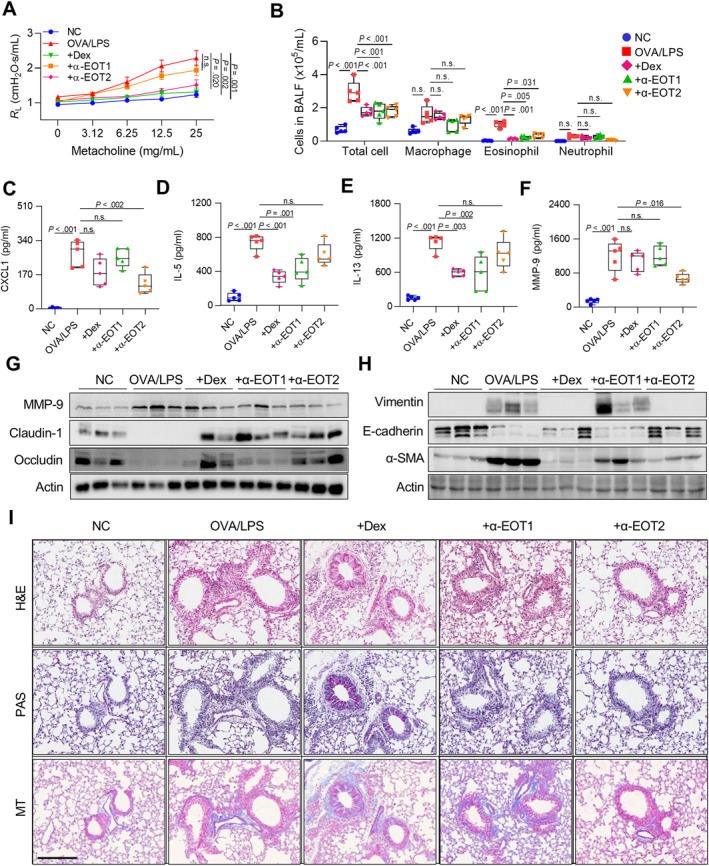
Regulation of airway inflammation and remodeling by neutralizing eotaxins in asthmatic mice. (A) Airway hyperresponsiveness. (B) Differential cell counts. The levels of (C) CXCL1, (D) IL‐5, (E) IL‐13, and (F) MMP‐9 in BALF. Data are presented as box plots, *n* = 5. *p* values were obtained by one‐way ANOVA with Bonferroni's post hoc test. Expressions of (G) MMP‐9 and tight junctions and (H) adherent junctions in the lung tissues. (I) Histological analysis of lung tissues stained with H&E, PAS, and MT. Scale bar, 200 μ.m.

Given that treatment of EOT1 and EOT2 induced distinct immune responses, neutralization against EOTs was conducted using specific antibodies, and the effects were compared with those of Dex in asthmatic mice. As a result, AHR was significantly decreased by anti‐EOT2 and Dex but not by anti‐EOT1 (Figure 5A). Additionally, all 3 treatments significantly decreased total cell and eosinophil counts, whereas no differences were found in macrophage or neutrophil counts in BALF (Figure 5B). Moreover, anti‐EOT2 reduced CXCL1 and MMP‐9 levels, while anti‐EOT1 and Dex reduced IL‐5 and IL‐13 levels in BALF (Figure 5C–F). However, EOT2, not EOT1, increased TIMP‐1 production in asthmatic airways (data not shown). In lung tissues, anti‐EOT2 suppressed MMP‐9 expression, whereas anti‐EOT1 or Dex did not. Furthermore, anti‐EOT2 attenuated tight junction disruption and EMT in asthmatic murine lungs (Figure 5G and H). Although all 3 treatments suppressed immune cell infiltration, anti‐EOT2 substantially decreased epithelium thickness and collagen accumulation in lung tissues (Figure 5I). These findings suggest that blockade of EOT2 alleviates airway remodeling by inhibiting MMP‐9 production and regulating epithelial barrier function in asthmatic patients.

## 4 Discussion

To our knowledge, this is the first study to demonstrate distinct roles of EOT1 and EOT2 in promoting airway inflammation and remodeling in the pathogenesis of SA. Patients with SA exhibited higher serum levels of EOT1 and EOT2 than those with NSA. Notably, serum EOT1 levels showed positive correlations with eosinophilic inflammatory markers, including blood and sputum eosinophil counts and serum ECP levels. In contrast, serum EOT2 levels were positively correlated with serum MPO, MMP‐9, and TIMP‐1 levels and negatively correlated with FEV_1_ (%) values. These findings suggest that EOT1 contributes to increased eosinophilic inflammation, whereas EOT2 is associated with neutrophil activation and lung function decline in asthmatic airways.

Although EOTs are recognized as selective chemoattractants for eosinophils, recent studies have suggested that EOTs have additional physiological function beyond chemotaxis, including the induction of eosinophil and basophil activation and degranulation through binding to CCR3 on their surface [17, 18]. EOTs reportedly recruit other CCR3‐expressing immune cells, such as macrophages, basophils, mast cells, and Th2 cells [10]. Furthermore, several studies have shown that exposure to LPS or influenza A induces CCR3 acquisition in infiltrated neutrophils in the lungs during inflammation or infection. Notably, stimulation of CCR3 can attract and activate neutrophils by enhancing reactive oxygen species production and neutrophil extracellular traps formation at inflamed sites, which was mitigated by CCR3 antagonist [13, 19, 20]. In the present study, serum EOT2 levels showed a positive correlation with serum MPO levels in asthmatic patients. Additionally, EOT2, but not EOT1, increased MPO production in LPS‐stimulated neutrophils of patients with SA, suggesting that EOT2 promotes neutrophil activation in the airways and thus contributed to increased disease severity in asthmatic patients.

AECs act as both physical and immunologic barrier against inhaled environmental substances, which are pivotal for host defenses in the innate immune system. To maintain airway homeostasis, epithelial barrier functions are precisely regulated by cell‐cell junctional complexes, including tight junctions and adherent junctions, which control paracellular permeability and epithelial leakiness [21]. Consequently, barrier dysfunction associated with disrupted junctional complexes can increase allergen sensitization, infectious susceptibility, and airway remodeling, ultimately leading to persistent AHR and asthma exacerbations in asthmatic patients [22, 23]. Multiple studies have demonstrated that increased MPO in neutrophils contributes to disrupted barrier functions and increased epithelial permeability by degrading tight junction, such as occludin and ZO‐1, causing barrier dysfunctions and mucosal inflammation in the intestinal epithelium [24, 25]. Furthermore, activated neutrophils promote ECM deposition and induce EMT, leading to airway remodeling and tissue fibrosis in asthmatic airways [6, 7]. In particular, patients with SA showed more activated neutrophils and increased MMP‐9 production in the airways, which are refractory to corticosteroid treatment [26, 27]. In our in vitro experiment, a pathogenic cycle was identified in which increased EOT2 secretion from AECs induced MPO release in neutrophils, ultimately promoting increased MMP‐9 production and tight junction disruption in AECs. Additionally, in vivo studies demonstrated that EOT1 enhanced T2/eosinophilic inflammation, whereas EOT2 increased AHR, promoted tight junction disruption, and facilitated ECM deposition in the lungs of LPS‐induced asthmatic mice. Finally, EOT2 promoted MMP‐9 expression and collagen deposition in lung tissues, effects that were mitigated by neutralizing EOT2 using its specific antibody.

In conclusion, EOT1 appears to promote T2/eosinophil recruitment and activation, whereas EOT2 accelerates airway remodeling and lung function decline by activating neutrophils. These findings provide a new insight into the distinct roles of EOT1 and EOT2 in the pathogenesis of SA.

## Author Contributions

**Soyoon Sim:** investigation, visualization, writing – original draft. **Eun‐mi Yang:** formal analysis. **Yoo Seob Shin:** project administration. **Seon Beom Kim:** writing – review and editing. **Youngwoo Choi:** conceptualization, supervision, funding acquisition, writing – review and editing. **Hae‐Sim Park:** conceptualization, supervision, funding acquisition, writing – review and editing.

## Conflicts of Interest

The authors declare no conflicts of interest.

## Data Availability

The data that support the findings of this study are available from the corresponding author upon reasonable request.
